# Clinical Dashboard in the Intensive Care Unit: Need-Assessment and Survey about Attitudes and Acceptance of Tele-ICU from the Viewpoint of Nurses and Clinicians in the Intensive Care Unit

**Published:** 2019-02

**Authors:** Mehdi Mohammadi, Kambiz Bahaadinbeigy, Mehdi Ahmadinejad, Behrang Chaboki, Hamed Tabesh, Kobra Etminani

**Affiliations:** 1 Medical Informatics Department, Mashhad University of Medical Sciences, Mashhad, Iran,; 2 Medical Informatics Research Center, Institute for Futures Studies in Health, Kerman University of Medical Sciences, Kerman, Iran,; 3 Department of Anesthesiology and Critical care Medicine, Kerman University of Medical Sciences, Kerman, Iran,; 4 Computer Science Department, School of Mathematical Science, Vali-e-Asr University of Rafsanjan, Rafsanjan, Iran.

**Keywords:** Intensive care unit, telemedicine, Tele-ICU, Clinical dashboard, ICU staff

## Abstract

**Background:**

One of the most worrying aspects of medical area in developing countries is the Intensive Care Unit (ICU). This study aimed to evaluate the acceptability of the clinical dashboard by the users, prior to final use and their attitude towards this technology, as well as to examine the specific needs that Tele-ICU technology can cover in the form of a clinical dashboard.

**Materials and Methods:**

This study was conducted at Shahid Bahonar Hospital of Kerman, Southeastern Iran, with three ICUs, the first, second, and third sections of which had 10, 12, and 24 beds, respectively. Taking survey and need assessment of care providers, qualitative and quantitative analyses were undertaken to identify key positive and negative themes. The data were analyzed by SPSS software version 18.

**Results:**

About 82% of care providers in the ICU participated in this survey. The number of participants based on the groups in the survey was 98 (81.7%) of the nurses and respiratory therapists group, 20 (80%) from the group of anesthesiologists and 20 (87%) from the group of anesthesiologist assistants who participated in the survey. About 51% of the survey participants completed the description section either partially or totally. On average, among all groups, the group of anesthesiologists had the most and the nurses had the least knowledge about telemedicine and Tele-ICU, whereas the anesthesiologist assistants had the most and the nurses and respiratory therapists group had the least knowledge about clinical dashboards.

**Conclusion:**

This study showed that the level of knowledge and awareness of care providers, especially nurses and respiratory therapists in the ICU in terms of telemedicine and Tele-ICU is low and care providers are in doubt that telemedicine technology could have a positive or negative impact on human resource shortages, yet agreed that it would have a negative effect on the privacy of the patients and care providers. In addition, the ICU care providers agree that Tele-ICU can positively affect the quality of patient care, staff satisfaction, reduce the cost of care, and ease and reduce the time for patient counseling. This suggests the need for further research and education of system impact beyond patient outcomes related to this new technology.

## INTRODUCTION

One of the most worrying aspects of medical area in developing countries is the Intensive Care Unit (ICU) (1–3). Economic growth and the population ageing are two important factors that are causing the increasing demand for intensive care (2, 4). Iran is one of the countries that encounter the challenge of population aging and the increase of old population in the health care system (5). On the other hand, one of the main causes of ICU patient's hospitalization in Iran is road accidents (6). Considering the fact that the growth rate of road accidents in Iran is 20 times faster than that of the global average (7), and that Iran has the most road accidents in the world (8), it is necessary and inevitable to pay attention to the growth rate of hospitalized patients in ICUs in Iranian hospitals. Nurses and clinicians who work in the ICU are required to have more experience and skills than other staff who work in the health care system, mainly due to their work type and its sensitivity. Studies have shown that staff experience in the ICU will have a direct impact on the quality of patient care (9). On the other hand, the health care system in Iran suffers from a shortage of manpower, especially a trained work force, and the ratio of manpower to patient in the ICU of Iranian hospitals is much lower than the global average (10). Therefore, staffs in the ICU are encountering shortage of time due to the compactness of the work, and one of the ways to overcome this problem is to delegate some time-consuming, important, and supervising tasks to technology. One of these technologies in the field of health is telemedicine, which in the ICU area, can be interpreted as Tele-ICU.

Patients in the ICU need constant and continuous care and, given that in this section, the patients' data is increasingly being generated from various software and hardware resources, accurate monitoring of these data is very important and vital (11). One of the initiatives that can improve this monitoring is the use of Tele-monitoring tools. The remote technology used in the present study to monitor the patient's condition in the ICU is a clinical dashboard. The clinical dashboard is an integrated system and provides its users with relevant and timely information to help them make the right decisions in improving patient care. Accessing to several different data sources, in fact the clinical dashboard provides information in a visual, summary, concise, and high-end format (12). In the present study, we integrated data from two different data sources, namely, vital signs monitors and hospital information system in the ICU, and therefore, using a clinical dashboard, these data were provided to nurses and clinicians in the ICU. In fact, we asked them to examine the clinical dashboard instead of reviewing the patient's paper in order to evaluate the effect of the clinical dashboard on the quality of patient care. Dowding et al. in their article review examined the role of clinical dashboards focused on improving patient care (13). Also, studies have been conducted to examine the role of Tele-ICU on patient costs and patient outcome (14–24). Some of these studies have mentioned some advantages for the use of Tele-ICU (15–18, 20–24), yet some of the recent prospective studies have not had a significant impact on patients' outcome (14, 19). Many of the Tele-ICU technologies used in these studies have been developed by commercial vendors and one of the limitations of these products is the lack of attention to the role and benefits of the patient in terms of using. One of the limitations of such products is the lack of personalization and lack of attention to the specific needs of care providers in using them, so that important aspects such as patients' privacy, data confidentiality, reliability, and trust in the integrity of product results are neglected and/or not properly addressed (25). Another obstacle to the use of Tele-ICU technologies mentioned in the previous studies is the reluctance and resistance of care providers in using them (15, 21). Generally, the user's consent and motivation in using technology is mentioned as one of the main factors of the success of IT projects (26). Shahpori et al. used a survey tool in their study to examine the attitudes of care providers in the ICUs toward Telemedicine (27). Since we used the clinical dashboard as a telemedicine tool in the ICU and in this study it is presented in the form of a website, therefore, our goal was firstly, to evaluate the knowledge and awareness of care providers about telemedicine and Tele-ICU in the ICU; secondly, to assess the acceptability of clinical dashboard in the ICU by care providers; and thirdly, to assess the need for services that clinical dashboards are supposed to offer to care providers in the future.

## MATERIALS AND METHODS

### Research environment and study setting

This study was conducted at Shahid Bahonar Hospital of Kerman, the center of trauma in southeastern Iran. The center included three ICUs, the first, second, and third sections of which had 10, 12, and 24 beds, respectively. Also, the bed occupancy rates in these three sections were respectively 96, 94, and 92% at the end of September 2017. Generally, patients admitted in these three sections were multi-service trauma patients with an average APACHE score of 19. The ratio of nurses to patients in any part of a day was 2:1. The total numbers of nurses employed in these three sections was 110 and the numbers of respiratory therapists and anesthesiologists were 10 and 25, respectively. Also, due to the educational nature of the center, 23 anesthesiologist students were involved in these three areas. The average work experience of nurses was 7 years and older (Table 1).

**Table 1.** Characteristics of ICU staff and clinicians

**Care givers**	**Male**	**Female**	**Work experience Average**	**Average age**
Anesthesiologists	17	8	10	46.1
Nurses	14	96	7.1	32
Anesthesiologist assistant(student)	9	14	2.6	35.3
Respiratory therapists	2	8	8.2	34.3
Total	42	126	7	34.7

Taking survey and need assessment of care providers, a questionnaire can be designed and provided to care providers to complete it and its results are reviewed. In this study, a survey tool that was used for the first time in the study of Shahpori, et al. (27) was employed. The survey instrument based on the circumstances of the case study was edited and modified. The questionnaire items were a combination of open-ended and clear questions. First, the questionnaire was translated from English into Persian. Then, in order to evaluate the cross-cultural comparison of the translation, the person who was knowledgeable in English proficiency retranslated the text once more into English to check whether that was equivalent to the original one. To assess the validity of the questionnaire, we conducted the following steps: we provided it to 10 individuals with medical informatics expertise who were familiar with telemedicine and Tele-ICU. Using the opinions of this group, the validity of the questionnaire was confirmed. The clinical dashboard was evaluated from the viewpoint of usability, prior to being presented to care providers and its problems were resolved.

### Participants

In this study, 138 care providers from three groups were invited to participate in the survey. The characteristics of the participants are shown in Table 1.

1. Nurses and respiratory therapists who have had at least 6 years of work experience in ICUs (t= 98).
2. Anesthesiologists who have had at least 9 years of work experience in the ICU (t= 20).
3. Anesthesiologist assistants (students) who have had at least 2 years of work experience in the ICU (t= 20).

In the present paper, all care providers involved in the ICUs of the study area were included in the study and, therefore, the sampling method was census.

### Preparation and data collection

Due to the fact that the concept of telemedicine and Tele-ICU were almost unknown among care providers, and they had little knowledge and awareness about it, and in order to prevent the growth of the rate of uncompleted or poorly-completed questionnaires (and resultantly, the distortion of the study findings and results), it was decided, based on the suggestion of experts in this field, that a general training session on a basic introduction to telemedicine and Tele-ICU be held for the care providers before the survey was conducted.

Thereupon, with an aim to get the the participants acquainted with telemedicine and Tele-ICU, a general training session was held for the care providers. The duration of the training session was 60 minutes, and the contents included the initial familiarity with the concepts of telemedicine, the concept of Tele-ICU, the concept of the clinical dashboard, the general purpose of conducting a survey, and how to complete a questionnaire. The subjects presented with the content of telemedicine and Tele-ICU, in terms of training the participants, were adapted from the educational and guideline categories of the field of anesthesia and intensive care, these materials were collected with the opinion of experts in this field (i.e. the second and third authors) (28, 29). One week after the initial training session, a questionnaire was given to the participants according to a timetable, to get completed and delivered. In addition, the participants who were interested to have an interview completed and delivered the questionnaires in a face-to-face meeting. 10% of the participants completed the questionnaire during interviews, while 80% of the participants completed and delivered the questionnaire in sooner than one day, and only 10% of the participants requested more time than one day to complete the questionnaire and on average delivered the questionnaire after 5 days. Two of the participants delivered the questionnaire without a response and did not have willingness to cooperate in the study and were eventually excluded from the study. Completed forms were analyzed after being collected.

### Data analysis

Based on the characteristics of the questionnaire, the analysis of the study was carried out in three parts: quantitative, semi-quantitative, and qualitative. In order to analyze the quantitative part, the percentage of the participants in the survey were evaluated based on their age, gender, work experience in ICU, and their general knowledge about telemedicine, especially Tele-ICU. In order to complete the semi-quantitative section, we first categorized Likert scale responses into three categories of negative / no response (0 or 1), abstentions (response 2) and positive (responses 3 or 4). Based on this categorization, the responses of participants in different groups were recorded and analyzed. Chi-square test was used to test the differences among the responses. Also, P-value less than 0.05 which indicates the significant difference, was used in all tests of the present study. In the qualitative analysis section, all participants' speeches were extracted and categorized according to the groups of participants. This was further investigated to find differences between the responses of different groups. Also, common topics and areas of the comments were extracted and the responses were categorized accordingly. To record the data and to perform the statistical analysis, Microsoft Excel 2010 and SPSS 18 software were utilized.

## RESULTS

About 82% of the care providers in the ICU participated in this survey. The number of participants based on the groups in the survey was 98 (81.7%) of the nurses and respiratory therapists group, 20 (80%) from the group of anesthesiologists, and 20 (87%) from the group of anesthesiologist assistants (students) who participated in the survey. About 51% of survey participants completed the description section either partially or totally. The group of anesthesiologists had the most and the nurses and respiratory therapists group had the least participation in the completion of the description section. In the privacy of care providers, the smallest number of participants attended in completing the description section (5%), and in the issue of reducing the cost of care, the largest number attended in completing the description section. On average, the group of anesthesiologists had the most, and the nurses had the least knowledge about telemedicine and Tele-ICU than the other groups. Anesthesiologist assistants had the most and the nurses and respiratory therapists group had the least knowledge about clinical dashboards (Table 2).

**Table 2.** Participants’ knowledge about telemedicine, Tele-ICU and clinical dashboard

**Care Providers**	**Telemedicine knowledge mean(±SD)**	**Tele-ICU knowledge mean(±SD)**	**Clinical dashboard knowledge mean(±SD)**
Anesthesiologists	2.2(1.0)	2.5(0.8)	1.6(1.0)
Nurses and Respiratory therapists	1.7(1.0)	1.4(0.9)	1.5(1.0)
Anesthesiologist assistant(student)	1.7(0.9)	2.0(0.9)	2.6(0.8)

On average, between 13% to 68% of the participants selected at least one answer as 'no opinion' in the questionnaire. There was no significant difference in the number of responses with the title “I have no idea” among the groups of the participants. Also, the largest number of responses with the title “I have no idea” was in the group of nurses and respiratory therapists (60%) and in the section affecting the shortage of specialized human resources and the least was in the group of Anesthesiologist assistants (2%) and was in the section on the ease and reduction of time for patient consultation. In addition, the highest numbers of positive responses were in the section of reducing the cost of care (76%) and the highest negative responses / no change were in the privacy of patients (64%) (Table 3).

**Table 3.** The effect of Tele-ICU on Care aspects

**Questions of the Survey**	**Negative/no change**	**I don’t have idea**	**Positive**	**Completing the comment section**	**Leaving the comment section**
Lack of expert human resources	18(13%)	94(68%)	26(19%)	10(7%)	128(93%)
Quality of care	18(13%)	24(17%)	96(70%)	55(40%)	83(60%)
Satisfaction of the patient and his/her relatives	35(25%)	44(32%)	59(43%)	23(17%)	115(83%)
Satisfaction of care providers	14(10%)	22(16%)	102(74%)	58(42%)	80(58%)
Transferring knowledge between experts	40(29%)	47(34%)	51(37%)	28(20%)	110(80%)
Patient privacy	88(64%)	28(20%)	22(16%)	15(11%)	123(89%)
Privacy of care providers	75(54%)	32(23%)	31(23%)	5(4%)	133(96%)
Reducing the cost of care	14(10%)	19(14%)	105(76%)	71(51%)	67(49%)
Decreasing patients visiting hours	48(35%)	48(35%)	42(30%)	33(24%)	105(76%)
Easiness and reducing time for patient consultation	26(19%)	18(13%)	94(68%)	47(34%)	91(66%)
Patient and his/her relative acceptance	47(34%)	62(45%)	29(21%)	18(13%)	120(87%)
Care provider acceptance	61(44%)	30(22%)	47(34%)	10(7%)	128(93%)

The findings showed that participants were in doubt about the positive or negative effects of Tele-ICU on the lack of skilled human resource, and, according to this case, significantly agreed on “I have no idea” (68%). At this stage, in the complementary opinions section, a group of participants, mostly from the nurses and respiratory therapists, were worried about the use of this technology and its negative impact on the lack of specialized human resources. In fact, they stated that the use of technology in the field of telemedicine in the ICU leaves the specialist human being lazy and away from the patient's bedside (Table 4).

**Table 4.** The comments and suggestions of the participating groups on the use of clinical dashboard in intensive care unit

**Questions in the survey and need assessment**	**Suggestion part**	**Complementary group**	**Descriptions**
**Lack of expert human resources**	10(7%)	Nurses and respiratory therapists	Laziness of expert human resources taking expert human resources away from the patient's bedside
**Quality of care**	55(40%)	Physicians and assistants	Provide alerts and notices timely diagnosis of errors
		Nurses and respiratory therapists	Contact with a remote physician Announcement of changes in the position of patients through the system
**Satisfaction of the patient and his/her relatives**	23(17%)	All participants	Advice and service to patients discharged from the ICU remotely at home
**Satisfaction of care providers**	58(42%)	Nurses and respiratory therapists	Raising the accuracy and speed of care providers Reducing the mistakes of care providers
		Physicians and assistants	The absence of a physician on the patient's bedside in non-emergency cases The system is assisted decision making of the physicians
**Transferring knowledge between experts**	28(20%)	Assistants	Providing remote consultation among the experts
**Patient privacy**	15(11%)	All participants	Worrying about the data security due to the web based system of clinical dashboards The privacy of the patient and care providers as a result of using the camera to monitor the patient's condition
**Privacy of care providers**	5(4%)	All participants	Worrying about the data security due to the web based system of clinical dashboards The privacy of the patient and care providers as a result of using the camera to monitor the patient's condition
**Reducing the cost of care**	71(51%)	Nurses and respiratory therapists	Reducing the presence of a physician in the intensive care unit and, consequently, reducing patient costs Improving the management of resources, especially pharmaceutical resources
		Physicians and assistants	Management of changing the patient's condition at specified times to reduce bed sores
**Easiness and reducing time for patient consultation**	47(34%)	Nurses and respiratory therapists	Reducing the time taking consultation request till having it
		Physicians and assistants	Access to remote clinical dashboard, especially access to laboratory and pharmaceutical data of patients remotely

Concerning the effect of Tele-ICU on the quality of care, the findings showed that most participants (70%) agreed on the positive impact of this technology. In the complementary opinion section, most physicians and assistants reported that warnings and notices of the systems can help the care providers in some cases, such as drug interactions, drug sensitivities, specific conditions, limiting certain patients, diagnosis of some errors, and improving the quality of care. The group of nurses and respiratory therapists also focused on such issues as communication with a remote physician through this technology, the prevention of drug interactions, the monitoring of specific patients through a remote camera, and the announcement of changing the position of patients through the system as the positive effects of this technology (Table 4).

74% of the participants agreed that the technology would have a positive impact on the satisfaction of the care providers. Most of the cases in the nurses and respiratory therapists' comments generally emphasized that due to the intensive work in the ICU, the presence of IT technologies, by increasing the accuracy and speed of personnel performance and reducing their errors, can give them comfort and ease the processes which results in the satisfaction of the personnel. On the other hand, physicians and assistants reported that the absence of a physician on the patient's bedside in many cases due to the presence of clinical dashboards can facilitate the physician's comfort and satisfaction. Also, there are suggestions that the system could be used as an assisted decision-making to help the physician in many cases and to provide satisfaction to the physician's performance. The participants also suggested that if a system could be developed to support and remotely give advice and consultation to patients who are discharged from the ICU and need special services this could considerably satisfy the patients and their relatives (Table 4).

20% of the participants, all of whom were from assistants and only one physician was among them, had suggested that the system could provide a type of remote consultation among the physicians which help them to transfer the knowledge (Table 4).

Most participants with 54 and 64% agreed on the negative effects of Tele-ICU and clinical dashboard on the privacy of personnel and patients. In the comments section, participants were worried about the patient's data and information security due to the web-based system of the clinical dashboard. On the other hand, many opinions emphasized that the use of the camera to monitor the patient's situation threatens the privacy of personnel and patients (Table 4).

76% of the participants emphasized the positive impact of this technology on reducing the cost of care. In the comments section, the nurses and respiratory therapists group explained that due to the significant cost of the patients' expenses for the ICU must be paid to their physician, it is possible to use the remote technology to decrease the presence time of physicians in department and hence the cost of the patient will decrease. In this section, physicians and assistants emphasized that using a clinical dashboard can improve the management of resources, especially the medicinal resources, and prevent the loss. The group's suggestion was that management of changing the patient's condition at certain times could lead to a reduction in bed sores and would prevent high costs for the patient and the health system (Table 4).

68% of participants agreed on the positive effect of clinical dashboard on ease and reducing the time for patient consultation. In the comments section, two remarkable comments and suggestions were presented: the first comment, presented by a group of nurses and respiratory therapists, complained about the time-taking period of request for counseling till having a physician's face-to-face consultation, and given that the counselor physician often examines the patients' data, the counseling time will be dramatically reduced by accessing to patients' data remotely. The group of physicians and assistant also suggested the access to a remote clinical dashboard and said that access to laboratory and drug data from remote patients could facilitate the clinical counseling (Table 4).

## DISCUSSION

Telemedicine in the ICU is presented as a potential to overcome the challenges ahead and providing a range of special services with a desirable quality to the patients and partly compensate for the lack of expert human resources (30). Although recent studies in the US claimed that the development of Tele-ICU can lead to improving the patient outcomes (15–18 , 20–24), yet recent studies have not shown the evidence of the benefits of this technology to the patient's outcomes (14, 19). However, the findings of this study showed that care providers believed that using remote technology can improve a number of patient outcomes, including quality of patient care and patient safety, and, in some cases, reduce the patient costs. There is almost no published report that can prove the positive impact of Tele-ICU implementation on the challenge of the shortage of specialized human resources, and in this study, participants also had doubts about the fact that whether it could have a positive or negative effect. There are also reports of staff disagreements with the Tele-ICU implementation (19, 31). There is still no article published on the study of the cultural acceptance of staff, especially ICU physicians, for the implementation of Tele-ICU.

One of the benefits of our study was to measure the level of knowledge of care providers. As the results of the study indicated that the level of knowledge of the care providers about telemedicine, Tele-ICU, and clinical dashboard was low, which future plans for raising the knowledge can be designed.

Regarding using Tele-ICU in the study, we were able to provide a new feature for more patients monitoring, which would increase the patient safety and quality of care, especially if the system is in the ICUs of less developed areas. Also, according to the suggestions of participants in this survey, we can develop a remote consulting system alongside the clinical dashboard which will help to further promote and further medical knowledge.

Two important issues in this study expressed concern about the Tele-ICU system: the first problem was laziness of care providers due to the use of clinical dashboard information, which results in taking away from the patient's bedside, which is not pleasant; the second concern was the privacy of the patients and care providers in using this technology, which must be addressed and resolved. Another aspect to be addressed is the legal and security issues through which the patient data and their images are transmitted on the web and should be considered in order to enhance the security of this information and create a secure and appropriate context.

The purpose of this study was to provide a relatively unique evaluation of telemedicine and Tele-ICU before its implementation and deployment in one of the most critical medical areas. To prevent the opposition from the staff and to increase its acceptance by the care providers, patients, and relatives, necessary measures must be considered. Although before implementing dashboard clinical in the ICU, the attitudes were measured, yet this does not mean that after its implementation, the system will work successfully, and will follow all goals set; the only thing that is important was the publication of these experiences and taking advantage of them in deploying the system as much as possible.

Due to the fact that the concept of telemedicine and Tele-ICU were almost unknown among care providers, and they had little knowledge and awareness about it, and in order to prevent the growth of the rate of unanswered questions in the questionnaire, according to the experts in this field, it was decided that a general training session in the field of telemedicine and Tele-ICU be held before the survey was conducted, and this was one of the limitations of the present study because it may affect the results of the survey. Another limitation of the study was the lack of participation of patients and their relatives in the survey. Also, this survey was conducted only in the ICU of a hospital located in the center of the province with the highest level of care, and this was another limitation of the study. The strengths of this study were the large number of participants in the survey, considering different groups of care providers in the ICU, considering new technologies and innovation, and their impact on various aspects in the field of health and intensive care.

## CONCLUSION

This study showed that the level of knowledge and awareness of care providers, especially nurses and respiratory therapists in the ICU in terms of telemedicine and Tele-ICU was low. Also, the results of the study showed that care providers were in doubt that telemedicine technology could have a positive or negative impact on human resource shortages, yet agreed that it would have a negative effect on the privacy of the patient and care providers. In addition, the results of the study showed that ICU care providers agreed that Tele-ICU can positively effect on the quality of patient care, staff satisfaction, reduce the cost of care, and ease and reduce time for patient counseling.

In subsequent studies, it is recommended that the questionnaire be conducted in the ICU of several hospitals in different cities with different levels of facilities. We also need to focus on acceptance of this technology by the patients and care providers before we plan to implement any Tele-ICU projects, and secondly, to develop a program for training and evaluation of the personnel in the Tele-ICU field in terms of increasing their knowledge and awareness.

## References

[1] Gomersall CD Critical care in the developing world - a challenge for us all. Crit Care 2010;14(2):131. 20346092 10.1186/cc8871 PMC2887112

[2] Adhikari NK Fowler RA Bhagwanjee S Rubenfeld GD Critical care and the global burden of critical illness in adults. Lancet 2010;376(9749):1339–46. 20934212 10.1016/S0140-6736(10)60446-1 PMC7136988

[3] Dünser MW Baelani I Ganbold L A review and analysis of intensive care medicine in the least developed countries. Crit Care Med 2006;34(4):1234–42. 16484925 10.1097/01.CCM.0000208360.70835.87

[4] Angus DC Kelley MA Schmitz RJ White A Popovich J Jr Committee on Manpower for Pulmonary and Critical Care Societies (COMPACCS) Caring for the critically ill patient. Current and projected workforce requirements for care of the critically ill and patients with pulmonary disease: can we meet the requirements of an aging population? JAMA 2000;284(21):2762–70. 11105183 10.1001/jama.284.21.2762

[5] Noroozian M. The elderly population in iran: an ever growing concern in the health system. Iran J Psychiatry Behav Sci 2012;6(2):1–6. PMC3940007 24644476

[6] Amininasab SS Azimi Lolaty H Moosazadeh M Shafipour V The relationship between human dignity and medication adherence in patients with heart failure. J Med Ethics Hist Med 2017;10:5. 29291038 PMC5746662

[7] https://www.unicef.org/iran/media_4783.html.

[8] http://www.worldlifeexpectancy.com/iran-road-traffic-accidents.

[9] Angus DC Kelley MA Schmitz RJ White A Popovich J Jr. Committee on Manpower for Pulmonary and Critical Care Societies (COMPACCS) Caring for the critically ill patient. Current and projected workforce requirements for care of the critically ill and patients with pulmonary disease: can we meet the requirements of an aging population. JAMA. 2000; 284(21):2762–70. 11105183 10.1001/jama.284.21.2762

[10] World Health Organization World Health Statistics 2015. Geneva: World Health Organization; 2015. World health statistics. 2016;2012.

[11] De Georgia MA Kaffashi F Jacono FJ Loparo KA Information technology in critical care: review of monitoring and data acquisition systems for patient care and research. ScientificWorldJournal 2015;2015:727694. 25734185 10.1155/2015/727694 PMC4334936

[12] Daley K Richardson J James I Chambers A Corbett D Clinical dashboard: use in older adult mental health wards. The Psychiatrist 2013;37(3):85–8.

[13] Dowding D Randell R Gardner P Fitzpatrick G Dykes P Favela J Dashboards for improving patient care: review of the literature. Int J Med Inform 2015;84(2):87–100. 25453274 10.1016/j.ijmedinf.2014.10.001

[14] Thomas EJ Lucke JF Wueste L Weavind L Patel B Association of telemedicine for remote monitoring of intensive care patients with mortality, complications, and length of stay. JAMA 2009;302(24):2671–8. 20040555 10.1001/jama.2009.1902

[15] From a distance: saving lives through remote care A combination of eICU technology, intensivists and in-house staffing delivers significant improvements in ICU patient care. Health Manag Technol 2007;28(3):26–9. 17425252

[16] Zawada ET Jr Kapaska D Herr P Aaronson M Bennett J Hurley B Prognostic outcomes after the initiation of an electronic telemedicine intensive care unit (eICU) in a rural health system. S D Med 2006;59(9):391–3. 17058472

[17] Leong JR Sirio CA Rotondi AJ eICU program favorably affects clinical and economic outcomes. Crit Care 2005;9(5):E22. 16277706 10.1186/cc3814 PMC1297635

[18] Shaffer JP Johnson JW Kaszuba F Breslow MJ Remote ICU management improves outcomes in patients with cardiopulmonary arrest.: 18. Critical Care Medicine 2005;33(12):A5.

[19] Morrison JL Cai Q Davis N Yan Y Berbaum ML Ries M Clinical and economic outcomes of the electronic intensive care unit: results from two community hospitals. Crit Care Med 2010;38(1):2–8. 19730249 10.1097/CCM.0b013e3181b78fa8

[20] Yoo EJ Dudley RA Evaluating telemedicine in the ICU. JAMA 2009 23;302(24):2705–6. 20040563 10.1001/jama.2009.1924

[21] Groves RH Jr Holcomb BW Jr Smith ML Intensive care telemedicine: evaluating a model for proactive remote monitoring and intervention in the critical care setting. Stud Health Technol Inform 2008;131:131–46. 18305328

[22] Breslow MJ Rosenfeld BA Doerfler M Burke G Yates G Stone DJ Effect of a multiple-site intensive care unit telemedicine program on clinical and economic outcomes: an alternative paradigm for intensivist staffing. Crit Care Med 2004;32(1):31–8. 14707557 10.1097/01.CCM.0000104204.61296.41

[23] Rosenfeld BA Dorman T Breslow MJ Pronovost P Jenckes M Zhang N Intensive care unit telemedicine: alternate paradigm for providing continuous intensivist care. Crit Care Med 2000;28(12):3925–31. 11153637 10.1097/00003246-200012000-00034

[24] Vespa PM Miller C Hu X Nenov V Buxey F Martin NA Intensive care unit robotic telepresence facilitates rapid physician response to unstable patients and decreased cost in neurointensive care. Surg Neurol 2007;67(4):331–7. 17350395 10.1016/j.surneu.2006.12.042

[25] Nguyen YL Kahn JM Angus DC Reorganizing adult critical care delivery: the role of regionalization, telemedicine, and community outreach. Am J Respir Crit Care Med 2010;181(11):1164–9. 20224067 10.1164/rccm.200909-1441CP

[26] Hwang MI Thorn RG The effect of user engagement on system success: a meta-analytical integration of research findings. Information & Management 1999;35(4):229–36.

[27] Shahpori R Hebert M Kushniruk A Zuege D Telemedicine in the intensive care unit environment--a survey of the attitudes and perspectives of critical care clinicians. J Crit Care 2011;26(3):328.e9–15. 10.1016/j.jcrc.2010.07.013 20869197

[28] Hanson C. William Medical Informatics. In: Ronald D Miller, editor. Miller’s Anesthesia. 8th ed Philadelphia: Elsevier Saunders; 2015 P 73–86

[29] Davis TM Barden C Dean S Gavish A Goliash I Goran S American Telemedicine Association Guidelines for TeleICU Operations. Telemed J E Health 2016;22(12):971–980. 27508454 10.1089/tmj.2016.0065

[30] LeapFrog Group Leadership group hospital survey. 2008 Available at http://www.leapfroggroup.org/media/file/2008_Survey_results_final_042909.pdf 2009.

[31] Parshuram CS Kirpalani H Mehta S Granton J Cook D Canadian Critical Care Trials Group In-house, overnight physician staffing: a cross-sectional survey of Canadian adult and pediatric intensive care units. Crit Care Med 2006;34(6):1674–8. 16625115 10.1097/01.CCM.0000218808.13189.E7

